# Supplementary material for: Growth and trend analysis of area, production and yield of rice: A scenario of rice security in Bangladesh
Source: PLoS One. 2021 Dec 10;16(12):e0261128. doi: 10.1371/journal.pone.0261128 (PMC8664192; doi:10.1371/journal.pone.0261128)
Supplement: Attachment — Submitted filename: RESPON~1.DOC [file pone.0261128.s003.DOC]

**Editor in Chief**

**Dear Sir,**

Thank you so much for handling our manuscript. We have tried incorporate all of your suggestions and comments into the manuscript. We also have responded the reviewers’ comments. We are also grateful for you and the reviewers to improve our manuscript.

**Response to academic editor comments**

**Comment 1:** Please ensure that your manuscript meets PLOS ONE's style requirements, including those for file naming.

**Response 1:** We have followed the manuscript requirement of PLOS ONE.

**Comment 2:** We suggest you thoroughly copyedit your manuscript for language usage, spelling, and grammar.

**Response 2:** We have edited our manuscript by the professional editor of Bangladesh Rice Research Institute. The details of the professional editor is

Md. Abul Kashem

Technical Editor

Publication and Public Relation Division

Bangladesh Rice Research Institute

Email: [head.pprd@brri.gov.bd](mailto:head.pprd@brri.gov.bd)

Edited copy of the manuscript has been uploaded as a “copyedit file” under supporting information section.

**Comment 3:** Data Availability statement

**Response 3:** The area, production and yield of rice data by seasons data from 1969-70 to 2019-20 is uploaded as a “data availability file” under supporting information section. We also revised data availability statement as per comments.

**Comment 4:** Copyright guidelines for figures

**Response 4:** GIS map describing the regional variation of area, production, yield and total rice were prepared by authors. However, administrative shape file of Bangladesh was downloaded and used from website of the Bangladesh Agricultural Research Council. Shapefile republished from Bangladesh Agricultural Research Council (BARC) database (http://maps.barcapps.gov.bd/index.php) under a CC BY license, with permission from Computer and GIS unit, BARC, original copyright 2014.

For the clarification of the copyright of maps, we added the above statements in figures 1, 4, 6 and 8 and “Data Used” section of materials and methods (Line: 127-131).

**Comment 5:** **Review of reference list to ensure that it is complete and correct**

**Response 5:** Reference has been corrected according to the PLOS ONE journal style. We have carefully checked in text citations.

According to the suggestions of technical editor we have added some new references in the manuscript and cited in the text accordingly.

**Response to reviewers’ comments**

Thank you for your insightful and valuables suggestions and comments for the improvement of our manuscript. We believe it helps to increase the scientific value of the manuscript and we are grateful for that.

**Comment 5.1:** The resolution of Figure 1, Figure 4, Figure 6, Figure 8, Figure 9 is too low to read clearly. It is necessary to provide high resolution of the above Figure.

**Response 5.1:** We have prepared and uploaded high resolution figures according to suggestions.

**Comment 5.2:** For Figure3(a), Figure11, Table2, Table3 and Table4, two-way-ANOVA is needed to analyze the data. A 2-way-ANOVA is a two-factorial analysis (here region/season and period as the 2 parameters). With suitable post-hoc test, this should be marked with distinct statistical groups (typically different alphabets) to allow one to see if there was difference in region/season and also between periods.

**Response 5.2:**

Thank you for your valuable comments for adding the scientific value of the manuscript. Figures 3(a) and 11 were revised accordingly. But in Table 2,3 and 4 represents the exponential growth rate which contains single observation. So far our knowledge, two-way ANOVA is not suitable for this type of growth data. Therefore, for understanding the statistical variations, we have incorporated the coefficient of variation instead of two-way ANOVA in tables 2,3 and 4.

If you have any idea for analyzing this type of data, please let us know.

**Comment 5.3:** The coefficient of variation for different regions is needed in the Table 4 to support the result “but region to region yield variations were very high” (Line 337 to 338).

Response 5.3: We are grateful to for your comments. We have added coefficient of variation in the Table 4.

**Comment 5.4:** In Figure12, for Aman and Boro most region have positive production growth rate. But, for Mymensingh, Bogura, Dinajpur, Rangpur, Dhaka and Faridpur have negative production growth rate where the other regions have positive production growth rate. 5.More detailed discussion is needed to illustrate this phenomenon.

**Response 5.4:**

We have added the following statements (Line: 460-468) according to the comments.

“We found most of the regions have positive production growth rate for Aman and Boro season. But in Aus season, especially Mymensingh, Bogura, Dinajpur, Rangpur, Dhaka and Faridpur have negative production growth rate. This is due to most of the aforementioned regions have dominated Boro-Fallow-T. Aman cropping pattern. In addition, those regions have intensified with non-rice cash crops and thereby the required growth duration of Aus is insufficient. Noticeably, the released Aus rice varieties are not easily fit into that cropping pattern. Besides, some regions have single Boro dominated cropping patterns due to adverse agro-climatic and geographical conditions. This is why some regions have negative production growth rate.”

**Comment 5.5:** More detailed discussion is needed to illustrate this phenomenon

**Response 5.5:** Discussion has been revised accordingly.

**Comment 5.6:** Minor revision: 2019-202 (Line201) should be revised as “2019-20”.

**Response 5.6:** The year is corrected accordingly.
